# Single-Center Experience of Using Liraglutide in Adolescents With Obesity +/- Type 2 Diabetes

**DOI:** 10.7759/cureus.58720

**Published:** 2024-04-22

**Authors:** Hajar Dauleh, Maheen Pasha, Hoda Gad, Basma Haris, Goran Petrovski, Houda Afyouni, Amal Khalifa, Saira Shehzad, Rasha Amin, Shiga Chirayath, Ghassan Mohamadsalih, Shayma Mohammed, Rayaz A Malik, Khalid Hussain

**Affiliations:** 1 Department of Pediatric Medicine, Division of Endocrinology, Sidra Medicine, Doha, QAT; 2 Department of Clinical Nutrition and Dietetics, Sidra Medicine, Doha, QAT; 3 Department of Research, Weill Cornell Medicine - Qatar, Doha, QAT; 4 Department of Research, Sidra Medicine, Doha, QAT

**Keywords:** childhood obesity, type 2 diabetes, pediatric endocrinology, liraglutide, obesity

## Abstract

Background

Childhood obesity is recognized as a chronic illness with limited therapeutic options. Tackling obesity (BMI; the weight in kilograms divided by the square of the height in meters, at the 95th percentile or higher) with lifestyle interventions, especially in adolescents, has proven to be a daunting task, yielding only modest results. Research on the use of liraglutide for weight reduction in pediatric patients has yielded conflicting results. Notably, there is a lack of studies in the Middle East reporting on the outcomes of glucagon-like peptide 1 (GLP-1) receptor agonists in treating obesity in children and adolescents, with or without diabetes. This study, conducted in the Middle East, represents the first investigation into the utilization of liraglutide for weight reduction in this pediatric population.

Methods

This retrospective study collected data on 22 consecutive participants, aged 12 to 19 years, who were diagnosed with obesity (defined as having a BMI greater than the 95th percentile for their age and sex) and had either type 2 diabetes mellitus (T2DM) or were non-diabetic who attended endocrine clinics in Sidra Medicine, Doha, Qatar, between 2020 and 2022. The study protocol involved a liraglutide treatment period spanning 18 months (72 weeks), with scheduled follow-up appointments at six-month intervals. The primary endpoints were changes in weight and BMI from baseline to the 72-week mark. Secondary endpoints were safety measures and changes in HbA1c.

Results

Out of the initial cohort of 22 patients, 12 completed the full 72-week duration of the study, while 10 patients either discontinued treatment or did not adhere to the prescribed medication regimen due to side effects. Among the 12 patients who completed the study, six had a diagnosis of T2DM. At baseline, the weight, standard deviation score (SDS), BMI, and BMI standard deviation (SD) were 113.9 kg, 2.9, 40.9 kg/m^2^, and 2.6 respectively. At the 18-month follow-up, the weight, SDS, BMI, and BMI SD were 117.8kg, 2.6, 39kg/m^2^, and 2.5, respectively. Thus, no statistically significant change in the weight parameters was evident at 18 months compared to baseline. Dropout from the study and poor compliance were high (10 out of 22 patients) due to side effects, mainly gastrointestinal (nausea, abdominal pain, diarrhea, and vomiting). No statistically significant differences were observed between obese vs. obese with T2DM. No significant change in HbA1c was found between baseline and treatment follow-up in the diabetes patients. No adverse effects in terms of impairment of liver and kidney function or pancreatitis were observed.

Conclusions

The administration of liraglutide to adolescents with obesity, regardless of whether they had T2DM or not, in a real-life setting, did not yield statistically significant reductions in BMI/weight parameters, and HbA1c levels at the 72-week mark. Nevertheless, the study findings indicate that liraglutide is deemed safe for utilization within this age group, despite the presence of mild gastrointestinal side effects.

## Introduction

The growing epidemic of childhood obesity has become a global public health concern. In 2022, 27 million children under the age of five and 390 million children and adolescents aged five-19 years were overweight, with an estimated total of 160 million living with obesity during the same period [1]. Over the past four decades, mean BMI and obesity in children and adolescents aged five-19 years have increased in most regions and countries [2,3]. Over the past 40 years, the expansion of obesity cases has escalated rapidly [2,3]. A 10-fold increase in the number of obesity cases among girls (from five million in 1975 to 50 million in 2016) and a 12-fold increase in the number of boys with obesity (from six million in 1975 to 74 million in 2016). At a local level, a cross-sectional study of school students living in Qatar in 2015-2016 revealed a prevalence of 44.8% and 40.4% among males and females, respectively, and 45.6% and 40.9% among Qatari and non-Qatari students, respectively [4].

The recognition of obesity as a chronic illness in 1998 by the National Institutes of Health (NIH) was a huge milestone in the fight against obesity [5]. This was followed in 2013 by the American Medical Association’s recognition of obesity as a complex, chronic disease that requires medical attention [5]. Long stigmatized as a reversible consequence of personal choices, obesity has complex genetic, physiologic, socioeconomic, and environmental contributors. As the environment has become increasingly obesogenic, access to evidence-based treatment has become even more crucial. Childhood obesity has been stigmatized as a reversible consequence of personal choices. However, based on accumulating evidence, obesity is a complex multifactorial process affected by environmental, socioeconomic, psychological, and genetic factors, especially since the environment has become increasingly obesogenic [6].

Screening, preventing, and early treatment of childhood high BMI is crucial due to its dramatic effects on children’s and adults’ health. Simmonds et al. reported that around 55% of obese children go on to be obese in adolescence; around 80% of obese adolescents will still be obese in adulthood, and around 70% will be obese over age 30 [7,8]. Also, obesity has caused an enormous burden on the healthcare sector. Excess body weight accounted for about four million deaths and 120 million disability-adjusted life-years worldwide in 2015 [9]. Nearly 70% of the deaths related to high BMI were due to cardiovascular disease, and more than 60% of those deaths occurred among obese individuals [10].

The risk of being overweight before puberty lies in the increased occurrence of premature male mortality, cardiovascular disease, and breast cancer death in females [8,9,10]. Various other conditions were identified in epidemiological studies, such as non-alcoholic fatty liver disease (NAFLD), diabetes mellitus, chronic kidney disease, obstructive sleep apnea, psychological issues, and musculoskeletal disorders. These conditions contribute to a range of functional limitations. Additionally, psychological effects and educational achievement are also important factors to consider [8,9,10].

The first-line treatment is a lifestyle modification that proved limited in achieving durable weight loss [11]. The only FDA-approved drugs in pediatrics are orlistat and phentermine, which have restricted efficiency in children younger than 12 years of age. Metformin is United States FDA-approved for use in children 10 years of age and older with type 2 diabetes mellitus (T2DM) and has been used off-label to achieve weight loss in children.

Saxenda (liraglutide), a glucagon-like peptide 1 (GLP-1) analog, obtained FDA approval in December 2020 for pediatric obesity treatment. This approval was grounded in the Satiety and Clinical Adipose Liraglutide Evidence (SCALE) Teens trial [12], specifically for chronic weight management in individuals aged 12 and older meeting obesity criteria. These criteria include specific BMI cut-offs for age and sex, corresponding to a BMI of 30 kg/m² or higher for adults and a weight exceeding 60 kg.

Liraglutide is an incretin analog and incretins are secreted naturally from the intestine in response to food ingestion and enhance insulin secretion to lower blood glucose levels. GLP-1 also slows gastric emptying and suppresses glucagon secretion, thus inducing a greater sense of satiety.

To date, no studies conducted in the Middle East have reported on the outcomes of GLP-1 receptor agonists in the treatment of obesity, both with and without diabetes, in children and adolescents. Consequently, our study represents the first investigation of liraglutide utilization for weight reduction in this population within the region. It is important to note that the longest treatment duration documented in the existing literature was 52 weeks. However, our study extends this timeframe to 72 weeks, enabling a more comprehensive assessment of the efficacy of liraglutide in real-life scenarios involving children and adolescents.

This article was previously presented as a poster at the 2023 European Society For Paediatric Endocrinology (ESPE) Annual Scientific Meeting on Sept 21, 2023.

## Materials and methods

Participants

In this retrospective study, data was collected on patients aged 12-19 with obesity, including those with and without T2DM, who attended endocrine clinics at Sidra Medicine, Doha, Qatar, between 2020-2022. Inclusion criteria included a BMI > 95th percentile for age and gender or a BMI z-score > 2. Exclusion criteria were type I diabetes mellitus, MODY (Maturity Onset Diabetes of the Young), monogenic obesity, history of pancreatitis, elevated calcitonin level, personal or family history of medullary thyroid cancer or MEN-2 (multiple endocrine neoplasia-2), abnormal liver or kidney function, recent heart disease, retinopathy or maculopathy, and recurrent severe hypoglycemia or hypoglycemic unawareness. Initially, twenty-two patients were included in the study, but ten dropped out during the study.

Study design

The study spanned 72 weeks of treatment with clinic follow-ups conducted at 24-week intervals (24 weeks, 48 weeks, and 72 weeks). The primary outcome measure was the change in BMI/weight measurements at 72 weeks compared to baseline. Secondary endpoints were safety measures and changes in HbA1c. 

Study procedure

Patient selection for liraglutide treatment was determined by primary physicians (pediatric endocrinology), following the hospital’s protocol. The initial phase involved gathering basic clinical and biochemical data, including fasting glucose, insulin, c-peptide, amylase, aspartate aminotransferase (AST), alanine aminotransferase (ALT), creatinine level, calcitonin, and family history of thyroid cancer. Baseline measurements of weight and related parameters such as BMI with Z-score were recorded. Patients were then referred to the endocrinology education clinic for guidance on liraglutide injection use, provided by a nurse educator. Education covered injection storage, disposal techniques, site rotation, and potential side effects. Families were provided with a titration protocol outlining weekly dose increments of 0.6 mg, aiming for a target dose of 3 mg. Dose adjustments were made based on side effects and treatment efficacy. Follow-up consultations conducted by physicians occurred at 24, 48, and 72 weeks, involving anthropometric measurements, inquiries about side effects and treatment adherence, dose titration, and lifestyle modifications. Blood tests including HbA1c, liver function tests, amylase, and creatinine level were performed at each visit. Customized dietary interventions tailored to patients' eating habits included incorporating low-fat, low-sugar snacks and emphasizing label reading for high-fiber and low-sugar options. Additional strategies such as the plate method, glycemic index awareness, and ensuring adequate fluid intake were discussed. Individualized exercise plans were developed based on preferences, offering options such as walking with a pedometer or engaging in age-appropriate sports.

Data analysis

Pairwise tables were analyzed using an Analysis of Variance (ANOVA) test. Subsequently, box plots were generated using R software (R Foundation, Indianapolis, Indiana, United States) to visually depict the data distributions.

## Results

A total of 22 participants who met the eligibility criteria were included in the study. Out of these, 10 subjects discontinued the treatment prematurely due to side effects, predominantly gastrointestinal symptoms such as nausea, vomiting, and abdominal cramps. The study was completed by 12 patients who demonstrated treatment adherence exceeding 80%. Among the participants, five were males and seven were females, with a mean age of 14.6 years and a median age of 15 years (range: 12-18 years). Six participants had a diagnosis of T2DM.

The average weight before intervention was 113.9 kg (standard deviation (SD) ± 22.7; range: 88.3 - 165.5) and the BMI was 41.5 kg/m² (SD ± 8.5; range: 23.1 - 52.9). The average standardized weight at presentation was 2.9 (SD ± 0.6; range: 2.16 - 4.11) and the standardized BMI was 2.6 kg/m² (SD ± 0.3; range: 2.11 - 3.07). At 24 weeks, the results indicated an average weight reduction of 3.8 kg, representing 3.36% of the initial body weight (3.36%; 95% confidence interval (CI): -7.8-0.21; p =0.06). 

**Figure 1:**
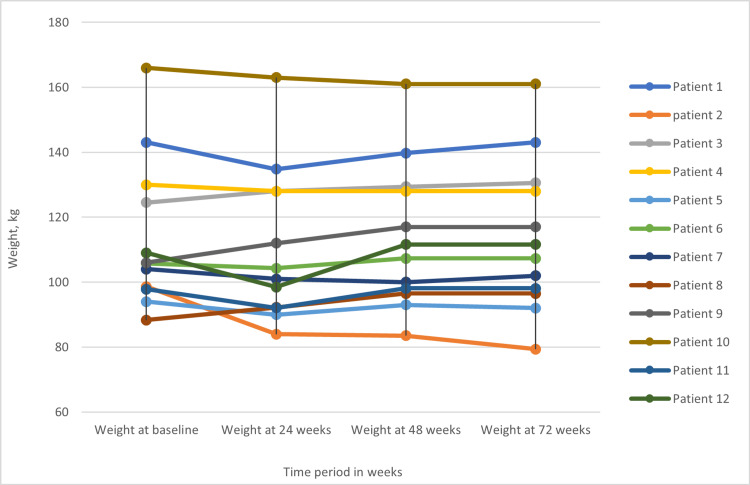
Changes in weight (kg) throughout the study showing a trend of initial weight reeducation at 24 weeks, followed by weight stabilization/increment.

However, the trend of weight reduction changed after the initial 24-week treatment period, as weight started to either increase or plateau (Figure 1).

**Figure 2:**
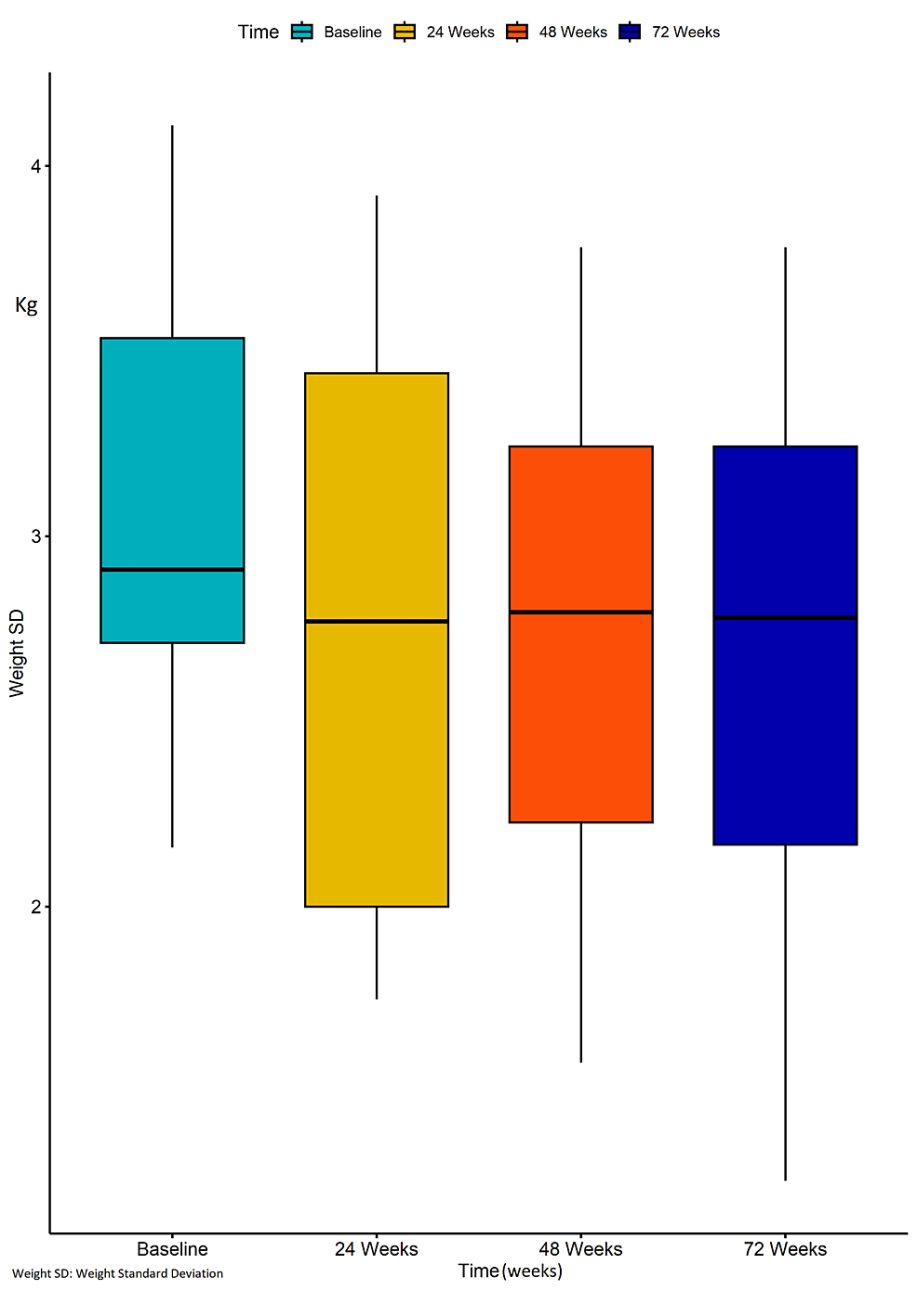
Boxplots of weighted standard deviation (WTSD) at baseline, 24 weeks, 48 weeks, and 72 weeks. Data were analyzed using a two-way repeated measures Analysis of Variance (ANOVA). No statistically significant difference was observed between the measures of WTSD at baseline and those at 24 weeks (p-value = 0.06). Similarly, there was no statistically significant difference between the measures of WTSD at baseline and those at 48 weeks (p-value = 0.11), as well as at 72 weeks (p-value = 0.09).

**Figure 3:**
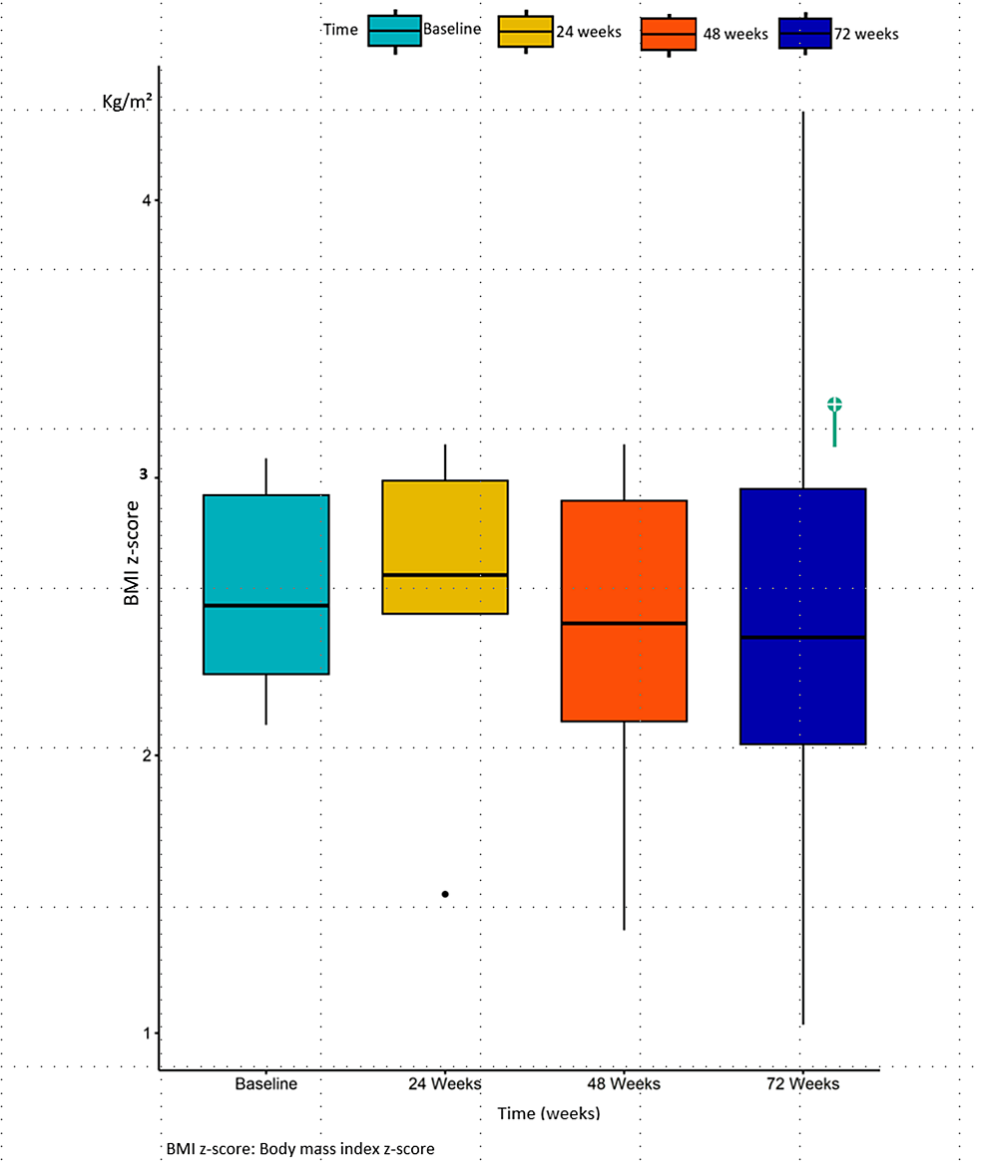
Boxplots of BMI z-score at baseline, 24 weeks, 48 weeks, and 72 weeks. Data were analyzed using a two-way repeated measures ANOVA. No statistically significant difference was observed between the measures of BMI z-score at baseline and those at 24 weeks (p-value = 0.752). Similarly, there was no statistically significant difference between the measures of BMI z-score at baseline and those at 48 weeks (p-value = 0.29), as well as at 72 weeks (p-value = 0.15)

By the end of the 72-week study period, the average weight was 115.3 kg (SD ± 34.9; range: 79.4-160.9) and the BMI was 39.54 kg/m² (SD ± 11.3; range: 24.5-51.6). There was no statistically significant change in the weight parameters at 72 weeks compared to baseline, as observed through pairwise comparison. Additionally, no statistically significant differences in weight reduction were observed between obese individuals and those with T2DM (Figures 2, 3).

**Figure 4:**
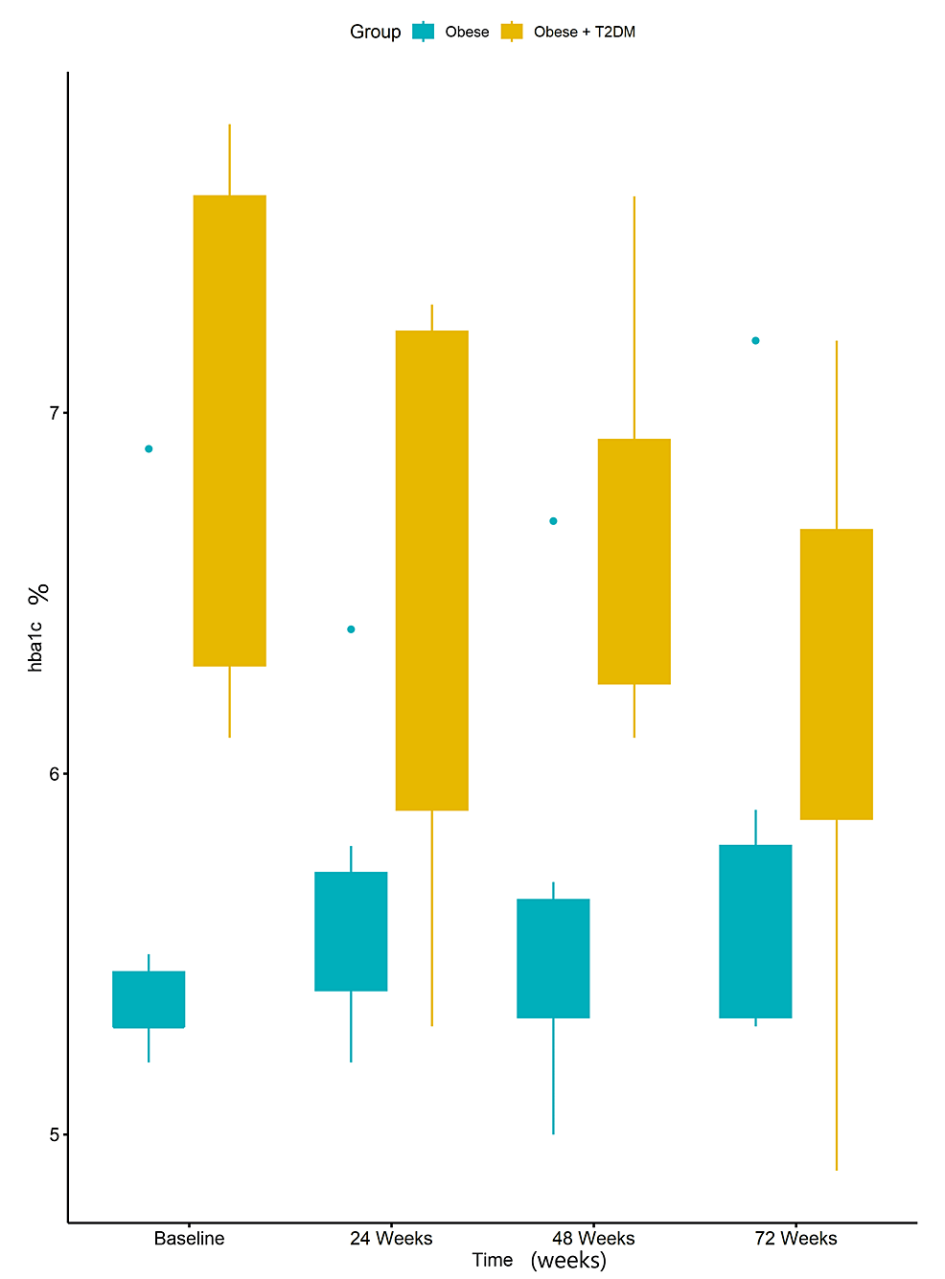
Boxplots of A1c of Obese + type 2 diabetes mellitus (T2DM) group vs Obese group at baseline, 24 weeks, 48 weeks, and 72 weeks. Data were analyzed using a two-way repeated measures Analysis of Variance (ANOVA). No statistically significant difference was observed between the measures of A1c at baseline and those at 24 weeks (p-value = 0.67). Similarly, there was no statistically significant difference between the measures of A1c at baseline and those at 48 weeks (p-value = 0.58), as well as at 72 weeks (p-value = 0.67).

At week 72, there was no substantial difference in the HbA1c compared to baseline in both treatment groups of Obese vs Obese with T2DM (Figure 4). 

**Figure 5:**
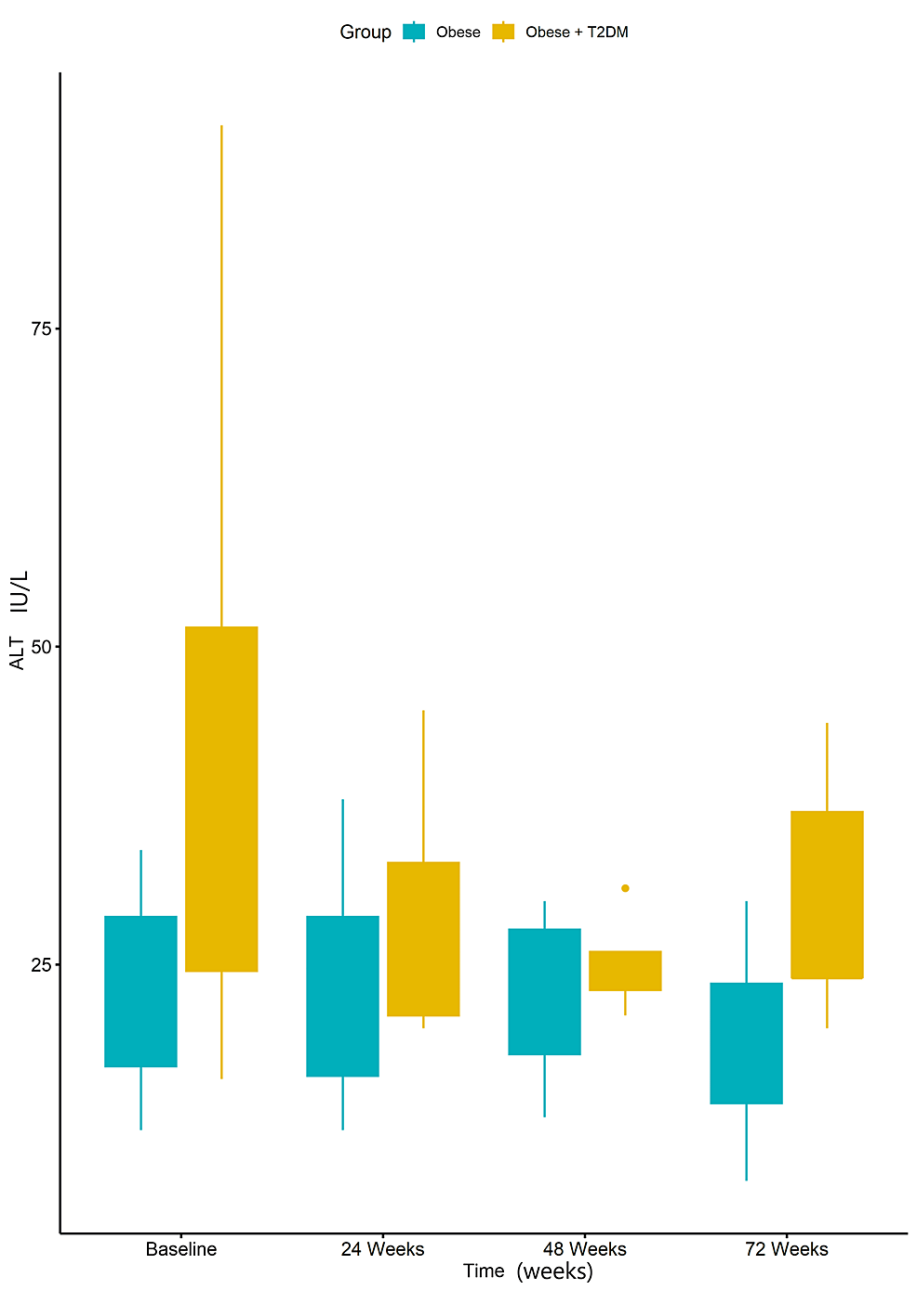
Boxplots of alanine aminotransferase (ALT) of Obese + type 2 diabetes mellitus (T2DM) group vs Obese group at baseline, 24 weeks, 48 weeks, and 72 weeks. Data were analyzed using a two-way repeated measures Analysis of Variance (ANOVA). No statistically significant difference was observed between the measures of ALT at baseline and those at 24 weeks (p-value = 0.617). Similarly, there was no statistically significant difference between the measures of ALT at baseline and those at 48 weeks (p-value = 0.201), as well as at 72 weeks (p-value = 0.393).

The baseline ALT levels in obese patients with T2DM were elevated and showed a tendency to decrease over the study period. However, these changes did not reach statistical significance (Figure 5). 

AST, creatinine, and amylase levels remained stable throughout the study, indicating the safety of liraglutide on the liver, kidneys, and pancreas, respectively. 

## Discussion

This study aimed to evaluate the efficacy and safety of liraglutide in adolescents with obesity, with or without T2DM, who attended endocrine clinics in Sidra Medicine between 2020 and 2022. Our real-life study showed no significant change in weight parameters at 72 weeks compared to baseline. The main phase 3 clinical trials comparing liraglutide to placebo reported variable outcomes in terms of reductions in BMI/weight parameters. In 2016, Danne et al. reported no statistically significant differences between the liraglutide group vs placebo in terms of BMI z-score and body weight [13]. Mastrandreal et al., 2019, reported a statistically significant reduction in BMI z-score in liraglutide-treated participants compared with the placebo group. A decrease was observed in body weight; however, it was not statistically significant [14].

Kelly et al. conducted the Satiety and Clinical Adipose-Liraglutide Evidence (SCALE) Teens trial in 2020 [12] and reported a significant BMI reduction in the liraglutide group in comparison to the placebo group at 56 weeks. A 5% decrease in BMI was observed in 43.3% of patients who used liraglutide, compared to only 18.7% of those who received a placebo. However, a greater increase in the BMI SD score was observed with liraglutide than with placebo after treatment discontinuation. Cornejo-Estrada, [15] reported in a meta-analysis of three clinical trials [12,13,14] that no statistically significant differences between liraglutide and body weight (kg; mean difference (MD): -2.62; 95% CI: -6.35 to 1.12; p = 0.17) and body mass index (kg/m2; MD: -0.80; 95% CI: -2.33 to 0.73, p = 0.31). However, it was shown that liraglutide might help reduce BMI and weight combined with a healthy diet and regular exercise. A lifestyle change may have favorable consequences that will be assessed in the future for adjunctive therapy [14].

For weight reduction in patients with T2DM 2, clinical trials were reported; Klein et al. [16] conducted a randomized controlled trial in 2014 on adolescents with T2DM. They reported stable mean body weight after the treatment period with liraglutide. In the Evaluation of Liraglutide in Pediatrics with Diabetes (Ellipse) 2019 trial, Tamborlane et al. [17] showed no difference in BMI z-score or body weight at week 26; however, his study involved patients with T2DM only. Several studies have been conducted at other centers examining the use of liraglutide. However, it is important to note that these studies were relatively short in duration and had small sample sizes. For instance, González-Ortiz et al. [18] conducted their study in Mexico over a period of 50 days and reported reductions in body weight, BMI, waist circumference, and adiposity among obese adolescents. Similarly, Kochar and Sethi [19] conducted a study in India lasting 12 weeks and involving 41 patients. Their findings revealed a significant decrease in body weight and BMI.

Patients exhibited a lack of adherence to lifestyle modifications, a crucial factor identified for achieving significant weight loss in previous research [20]. Despite conducting an initial consultation on lifestyle modifications, patients lost motivation to adhere to lifestyle interventions when weight loss was not observed.

Many participants displayed a significant psychological connection to food, highlighting the potential benefit of including a psychologist in the team. Apperley et al. [20] conducted a study with an intense weight management program with liraglutide. The participants were reviewed by a multidisciplinary team (MDT) every two weeks for advice and support and treated with daily subcutaneous injections of liraglutide (dose range 1.2-3.0 mg). The participants showed significant weight loss over the three months with an average reduction of 5.4 kg (4.2%; 95% CI: 1.93-8.78; p=0.0087). The mean drop in BMI was 2.1 kg/m2, which is statistically significant (95% CI: 0.973-3.199; p=0.0037). At the SCALE Teens 2020 [15], all participants received lifestyle therapy, defined as counseling on healthy nutrition and physical activity for weight loss.

Our study observed no statistically significant change in HbA1c levels among obese patients with T2DM in terms of glycemic control from baseline to the 72-week endpoint. It is important to consider, however, that previous literature reports have indicated the superior effectiveness of liraglutide compared to placebo in improving glycemic control specifically among youth with T2DM [19,20] The study has highlighted several limitations that need to be considered. The first limitation is the small sample size and single-center design, which may not provide a representative picture of the larger population. Secondly, the high dropout rate due to side effects suggests poor tolerability of the drug among adolescents with obesity, which may impact the effectiveness of the treatment. However, despite these limitations, the study provides valuable insights into the real-world use of liraglutide in treating adolescents with obesity. The findings emphasize the importance of an MDT approach that includes social workers, psychologists, dietitians, and exercise therapists to address the various aspects of patients' treatment. Furthermore, the high prevalence of adverse effects suggests the need for more staff to closely monitor patients, with monthly visits being recommended.

## Conclusions

In conclusion, liraglutide appears to be a viable treatment option for adults dealing with obesity and T2DM, yet its benefits for adolescents are somewhat limited. However, there is potential for improved outcomes when utilized in conjunction with an MDT approach and closely monitored follow-ups. The occurrence of gastrointestinal side effects in adolescents proved to be a significant drawback of the medication. To comprehensively evaluate the long-term efficacy and safety in this population, further studies with larger sample sizes and extended follow-up periods are warranted.
